# Re: Predictive and prognostic impact of preoperative complete blood count based systemic inflammatory markers in testicular cancer

**DOI:** 10.1590/S1677-5538.IBJU.2020.0103

**Published:** 2020-07-31

**Authors:** Cengiz Beyan, Esin Beyan

**Affiliations:** 1 Ufuk University Faculty of Medicine Department of Hematology Ankara Turkey Department of Hematology, Ufuk University Faculty of Medicine, Ankara, Turkey; 2 University of Health Sciences Kecioren Training and Research Hospital Department of Internal Medicine Ankara Turkey Department of Internal Medicine, University of Health Sciences, Kecioren Training and Research Hospital, Ankara, Turkey

*To the editor,*

We read with great interest the retrospective research of Arda et al. about the predictive and prognostic impact of some parameters based on complete blood count in patients with testicular cancer (1). The researchers suggested that some parameters such as neutrophil lymphocyte ratio (NLR), mean platelet volume (MPV) and red cell distribution width (RDW) could provide predictive and prognostic information in these patients. We would like to emphasize the factors that may have affected incorrectly some of the parameters evaluated in this study.

First, this study included retrospective data from two different centers of a very broad period of 10 years. Unlike prospective studies, it is not possible to reduce pre-analytical and analytical errors in retrospective studies.

MPV is a parameter which measurement methodology has not been standardized, which is significantly affected by epidemiological factors such as age, gender, race and variations in the measurement method (2). The age difference between the patient and the control group was a factor that may have affected the results in this study. It has been shown that the variability of the time elapsed from the venipuncture to the measurement time cause to deviations of 2-50% in the MPV results (3, 4). In addition, different technologies used for complete blood count also lead to deviations in results (4–6). MPV measurement times and devices used for MPV measurement were unknown in this study. All these factors make the MPV data of the study unreliable. The preoperative MPV cut-off value specified in Table 3 was probably incorrectly written, and actually, the fact that MPV measurement still could not been standardized today, it makes impossible to determine a cut-off value for MPV.

In the study, only lymphocyte and neutrophil percentages were used in comparisons and absolute lymphocyte and absolute neutrophil numbers were not specified. Since the white blood cell values of the cases are unknown and absolute lymphocyte and neutrophil numbers are not given, it remains to be not understood whether the statistical difference in the percentage of lymphocytes and neutrophils is a real difference between the patient and control groups. Moreover, the age difference between the patient and control groups was also statistically significant, and this was a condition that might have affected the results, as the absolute lymphocyte counts decrease with increasing age (7, 8).

It is stated in the discussion section that especially NLR and RDW can be used as predictive and prognostic factor with the highest sensitivity and specificity in patients with testicular cancer. However, in Table 4, where NLR 1.78 was used as the cut-off value, the fact that RDW values were not different between the groups with high and low NLR was a situation that did not support this statement.

As a result, some parameters based on complete blood count may have no predictive and prognostic impact in patients with testicular cancer.

## LIST OF ABBREVIATIONS

MPV: mean platelet volume
NLR: neutrophil lymphocyte ratio
RDW: red cell distribution width
